# *syn*-3-(4-Chloro­benz­yl)-1,5-dimethyl-3,7-diaza­bicyclo­[3.3.1]nonan-9-ol

**DOI:** 10.1107/S1600536812030310

**Published:** 2012-07-07

**Authors:** Konstantin V. Kudryavtsev, Sergey Z. Vatsadze, Vera S. Semashko, Andrei V. Churakov

**Affiliations:** a Department of Chemistry, M. V. Lomonosov Moscow State University, Leninskie Gory 1/3, Moscow 119991, Russian Federation; b Institute of Physiologically Active Compounds, Russian Academy of Sciences, Chernogolovka 142432, Moscow Region, Russian Federation; c Institute of General and Inorganic Chemistry, Russian Academy of Sciences, Leninskii prospekt 31, Moscow 119991, Russian Federation

## Abstract

In the title compound, C_16_H_23_ClN_2_O, both six-membered rings adopt chair conformations, thus allowing the formation of an intra­molecular N—H⋯N hydrogen bond. In the crystal, adjacent mol­ecules are combined into chains running along the *ac* diagonal *via* O—H⋯N hydrogen bonds.

## Related literature

For general background to chemistry affording *syn*-3,7-diaza­bicyclo­[3.3.1]nonan-9-ols, see: Vatsadze *et al.* (2006 ▶). 3,5,6,7-Tetra­substituted 3,6-diaza­bicyclo­[3.2.1]octa­nes, their biological activity as enzyme inhibitors and their X-ray structures have been reported by: Kudryavtsev (2010 ▶); Kudryavtsev & Churakov (2012 ▶).
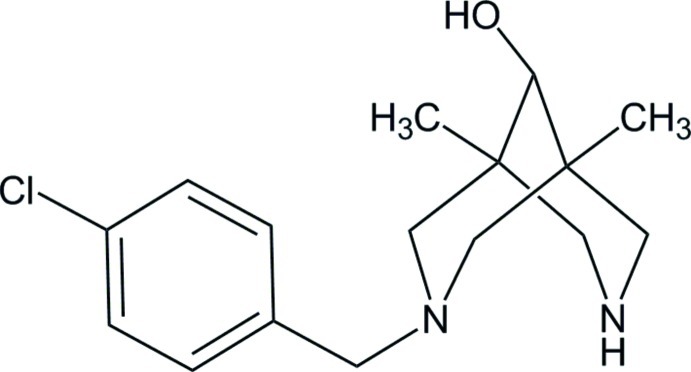


## Experimental

### 

#### Crystal data

C_16_H_23_ClN_2_O
*M* *_r_* = 294.81
Monoclinic, 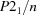

*a* = 7.9739 (4) Å
*b* = 16.8120 (9) Å
*c* = 12.1103 (6) Å
β = 107.520 (1)°
*V* = 1548.16 (14) Å^3^
*Z* = 4
Mo *K*α radiation
μ = 0.25 mm^−1^
*T* = 120 K
0.25 × 0.20 × 0.20 mm

#### Data collection

Bruker SMART 1K diffractometer
Absorption correction: multi-scan (*SADABS*; Bruker, 2008 ▶) *T*_min_ = 0.941, *T*_max_ = 0.953
10450 measured reflections
3747 independent reflections
2976 reflections with *I* > 2σ(*I*)
*R*_int_ = 0.034

#### Refinement

*R*[*F* ^2^ > 2σ(*F* ^2^)] = 0.042
*wR*(*F* ^2^) = 0.107
*S* = 1.02
3747 reflections
273 parameters
All H-atom parameters refined
Δρ_max_ = 0.39 e Å^−3^
Δρ_min_ = −0.36 e Å^−3^

### 

Data collection: *APEX2* (Bruker, 2008 ▶); cell refinement: *SAINT* (Bruker, 2008 ▶); data reduction: *SAINT*; program(s) used to solve structure: *SHELXTL* (Sheldrick, 2008 ▶); program(s) used to refine structure: *SHELXTL*; molecular graphics: *SHELXTL*; software used to prepare material for publication: *SHELXTL*.

## Supplementary Material

Crystal structure: contains datablock(s) I, global. DOI: 10.1107/S1600536812030310/ff2075sup1.cif

Structure factors: contains datablock(s) I. DOI: 10.1107/S1600536812030310/ff2075Isup2.hkl

Supplementary material file. DOI: 10.1107/S1600536812030310/ff2075Isup3.cml

Additional supplementary materials:  crystallographic information; 3D view; checkCIF report

## Figures and Tables

**Table 1:** Hydrogen-bond geometry (Å, °)

*D*—H⋯*A*	*D*—H	H⋯*A*	*D*⋯*A*	*D*—H⋯*A*
N2—H2⋯N1	0.870 (19)	2.287 (19)	2.8327 (18)	120.8 (16)
O1—H1⋯N2^i^	0.87 (2)	1.86 (2)	2.7242 (17)	172 (2)

Symmetry code: (i) 
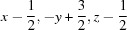
.

**Fig. 1.**
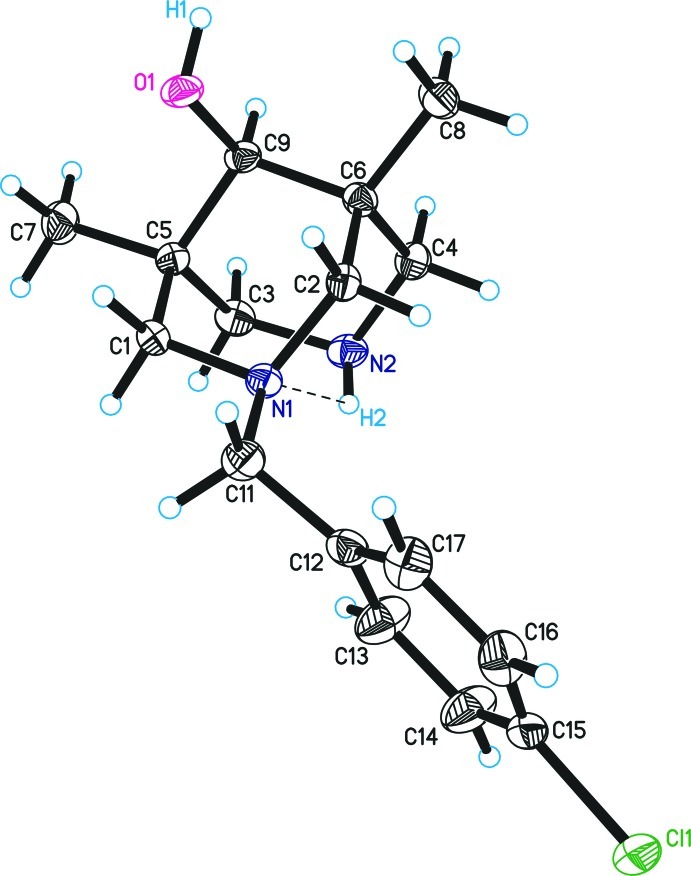
The molecular structure of the title compound, showing the numbering scheme adopted. Displacement ellipsoids are shown at the 50% probability level. Intramolecular hydrogen bond is drawn as dashed line.

**Fig. 2.**
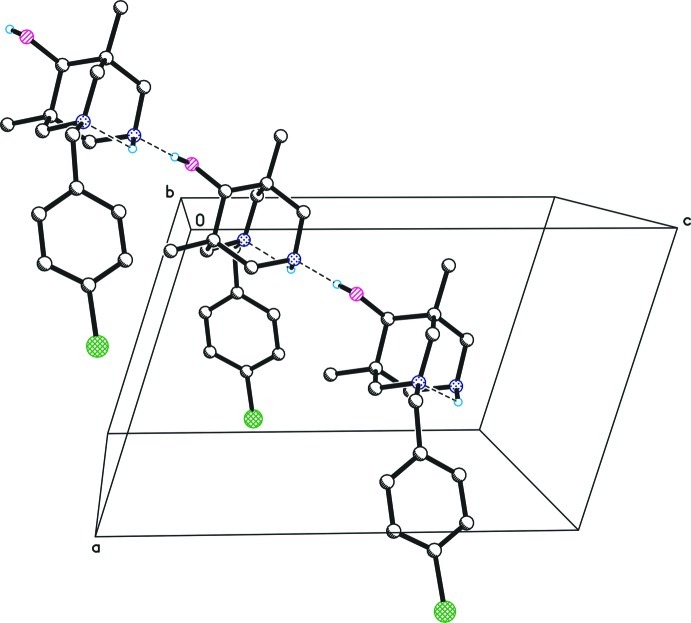
Hydrogen bonded chains spreads along ac-diagonal. Hydrogen bonds are drawn as dashed lines.

## supplementary crystallographic information

### Comment

In the title compound, both six-membered cycles adopt chair conformation (Fig. 1). The latter allows the formation of intramolecular N—H···N hydrogen bond. The hydroxyl group at the 9th position of the heterocyclic scaffold is situated closer to the tertiary N-atom. In crystal, the adjacent molecules are combined in chains running along *ac*-diagonal *via* O—H···N hydrogen bonds (Fig. 2).

*syn*-3,7-Diazabicyclo[3.3.1]nonan-9-ols are of interest as a structural motif for enzymes inhibitors. Inhibition of thrombin by substituted 3,6-diazabicyclo[3.2.1]octanes is reported (Kudryavtsev (2010)).

### Experimental

The solution of 2.23 g (6.9 mmol) of *anti*-5-(4-chlorobenzyl)-3-formyl- 1,5-dimethyl-3,7-diazabicyclo[3.3.1]nonan-9-ol (Vatsadze *et al.*(2006)) in conc. HCl (molar ratio *ca* 1:120) was refluxed for 21 h. The cooled solution was neutralized with NaOH to the pH 9–10. The precipitate formed was filtered off, washed with water to neutral pH of mother liquor, then once with ether, recrystallized from EtOH. *syn*-3-(4-Chlorobenzyl)-1,5-dimethyl-3,7-diazabicyclo[3.3.1]nonan-9-ol. Yield 1.50 g (74%), colourless crystals, sublimated at 473 K. ^1^H NMR (dmso-d_6_): δ 0.63 (s, 6H, 2CH_3_); 2.26–2.41 (m, 6H, H-6a, H-8a, H-4 e, H-4a, H-2 e, H-2a); 2.72 (d, 2H, H-6 e, H-8 e, *J* 13.3); 2.98 (d, 1H, H-9, *J* 4.5); 3.27 (s, 2H, CH_2_Ar); 4.74 (d, 1H, OH, *J* 5.0); 7.28, 7.38 (both d, both 2H, CH(3,5)(Ar), CH(2,6)(Ar), *J* 8.3). ^1^H NMR (dmso-d_6_ + CF_3_COOH): δ 0.77 (s, 6H, 2CH_3_); 2.36 (d, 2H, H-2a, H-4a, *J* 10.6); 2.48 (d, 2H, H-2 e, H-4 e, *J* 12.4); 2.86 (d, 2H, H-6a, H-8a, *J* 11.9); 3.21 (d, 2H, H-6 e, H-8 e, *J* 12.6); 3.24 (s, 1H, H-9); 3.43 (s, 2H, CH_2_Ar); 7.34–7.43 (m, 4H, H(Ar)). ^13^C NMR (dmso-d_6_ + CF_3_COOH): δ 20.3 (2CH_3_); 35.4 (C-1, C-5); 53.7 (C-6, C-8); 56.5 (C-2, C-4); 61.2 (CH_2_Ar); 73.4 (C-9); 117.4, 128.7, 131.7, 136.0 (C(Ph)). Anal. Calcd. for C_16_H_23_ClN_2_O: C, 65.20; H, 7.81; N, 9.51. Found: C, 65.52; H, 7.98; N, 9.35.

### Refinement

All hydrogen atoms were located in a difference Fourier map and refined isotropically.

### Crystal data

**Table:**

C_16_H_23_ClN_2_O	*F*(000) = 632
*M**_r_* = 294.81	*D*_x_ = 1.265 Mg m^−^^3^
Monoclinic, *P*2_1_/*n*	Mo *K*α radiation, λ = 0.71073 Å
Hall symbol: -P 2yn	Cell parameters from 4857 reflections
*a* = 7.9739 (4) Å	θ = 2.7–30.0°
*b* = 16.8120 (9) Å	µ = 0.25 mm^−^^1^
*c* = 12.1103 (6) Å	*T* = 120 K
β = 107.520 (1)°	Prism, colourless
*V* = 1548.16 (14) Å^3^	0.25 × 0.20 × 0.20 mm
*Z* = 4	

### Data collection

**Table:**

Bruker SMART 1K [or APEXII?]diffractometer	3747 independent reflections
Radiation source: fine-focus sealed tube	2976 reflections with *I* > 2σ(*I*)
Graphite monochromator	*R*_int_ = 0.034
ω scans	θ_max_ = 28.0°, θ_min_ = 2.1°
Absorption correction: multi-scan (*SADABS*; Bruker, 2008)	*h* = −10→9
*T*_min_ = 0.941, *T*_max_ = 0.953	*k* = −22→14
10450 measured reflections	*l* = −15→15

### Refinement

**Table:**

Refinement on *F*^2^	Primary atom site location: structure-invariant direct methods
Least-squares matrix: full	Secondary atom site location: difference Fourier map
*R*[*F*^2^ > 2σ(*F*^2^)] = 0.042	Hydrogen site location: difference Fourier map
*wR*(*F*^2^) = 0.107	All H-atom parameters refined
*S* = 1.02	*w* = 1/[σ^2^(*F*_o_^2^) + (0.0513*P*)^2^ + 0.4862*P*] where *P* = (*F*_o_^2^ + 2*F*_c_^2^)/3
3747 reflections	(Δ/σ)_max_ = 0.002
273 parameters	Δρ_max_ = 0.39 e Å^−^^3^
0 restraints	Δρ_min_ = −0.36 e Å^−^^3^

### Special details

**Table:**

Geometry. All e.s.d.'s (except the e.s.d. in the dihedral angle between two l.s. planes) are estimated using the full covariance matrix. The cell e.s.d.'s are taken into account individually in the estimation of e.s.d.'s in distances, angles and torsion angles; correlations between e.s.d.'s in cell parameters are only used when they are defined by crystal symmetry. An approximate (isotropic) treatment of cell e.s.d.'s is used for estimating e.s.d.'s involving l.s. planes.
Refinement. Refinement of *F*^2^ against ALL reflections. The weighted *R*-factor *wR* and goodness of fit *S* are based on *F*^2^, conventional *R*-factors *R* are based on *F*, with *F* set to zero for negative *F*^2^. The threshold expression of *F*^2^ > σ(*F*^2^) is used only for calculating *R*-factors(gt) *etc*. and is not relevant to the choice of reflections for refinement. *R*-factors based on *F*^2^ are statistically about twice as large as those based on *F*, and *R*-factors based on ALL data will be even larger.

### Fractional atomic coordinates and isotropic or equivalent isotropic displacement parameters (Å^2^)

**Table:**

	*x*	*y*	*z*	*U*_iso_*/*U*_eq_	
Cl1	0.97589 (5)	1.10266 (3)	0.39054 (4)	0.03592 (14)	
O1	−0.15380 (14)	0.82506 (6)	−0.00656 (9)	0.0220 (2)	
N1	0.16827 (16)	0.92965 (7)	0.19544 (10)	0.0176 (3)	
N2	0.22587 (17)	0.78671 (8)	0.32531 (11)	0.0211 (3)	
C1	−0.01659 (19)	0.92287 (8)	0.19298 (13)	0.0180 (3)	
C2	0.20407 (19)	0.88120 (9)	0.10373 (13)	0.0176 (3)	
C3	0.0379 (2)	0.79594 (9)	0.31288 (13)	0.0216 (3)	
C4	0.2528 (2)	0.75254 (9)	0.22011 (13)	0.0204 (3)	
C5	−0.07319 (18)	0.83646 (8)	0.20054 (12)	0.0173 (3)	
C6	0.14948 (19)	0.79358 (8)	0.10650 (12)	0.0168 (3)	
C7	−0.2661 (2)	0.83514 (11)	0.19815 (17)	0.0265 (3)	
C8	0.1851 (2)	0.74991 (10)	0.00522 (14)	0.0244 (3)	
C9	−0.04692 (19)	0.79066 (8)	0.09780 (12)	0.0173 (3)	
C11	0.2140 (2)	1.01297 (9)	0.18344 (15)	0.0224 (3)	
C12	0.4088 (2)	1.02924 (8)	0.23069 (14)	0.0212 (3)	
C13	0.4991 (2)	1.01062 (12)	0.34495 (15)	0.0332 (4)	
C14	0.6739 (2)	1.03189 (12)	0.39435 (16)	0.0350 (4)	
C15	0.7590 (2)	1.07151 (9)	0.32730 (15)	0.0249 (3)	
C16	0.6764 (2)	1.08801 (10)	0.21243 (16)	0.0284 (4)	
C17	0.5000 (2)	1.06683 (10)	0.16459 (15)	0.0267 (4)	
H1	−0.185 (3)	0.7863 (13)	−0.056 (2)	0.047 (6)*	
H2	0.275 (2)	0.8334 (12)	0.3320 (17)	0.029 (5)*	
H9	−0.082 (2)	0.7325 (11)	0.1019 (15)	0.024 (5)*	
H11	−0.029 (2)	0.9525 (9)	0.2591 (14)	0.014 (4)*	
H12	−0.097 (2)	0.9484 (10)	0.1210 (15)	0.022 (4)*	
H21	0.328 (2)	0.8846 (10)	0.1130 (14)	0.017 (4)*	
H22	0.144 (2)	0.9043 (10)	0.0278 (15)	0.018 (4)*	
H31	−0.013 (2)	0.7404 (11)	0.3163 (16)	0.027 (5)*	
H32	0.032 (2)	0.8250 (10)	0.3794 (16)	0.021 (4)*	
H41	0.219 (2)	0.6942 (10)	0.2163 (14)	0.017 (4)*	
H42	0.382 (2)	0.7540 (10)	0.2280 (14)	0.018 (4)*	
H13	0.438 (3)	0.9822 (12)	0.3919 (18)	0.041 (6)*	
H14	0.737 (3)	1.0203 (13)	0.4736 (19)	0.043 (6)*	
H16	0.736 (3)	1.1168 (14)	0.168 (2)	0.054 (7)*	
H17	0.438 (3)	1.0795 (12)	0.0842 (19)	0.043 (6)*	
H71	−0.343 (3)	0.8633 (12)	0.1288 (18)	0.040 (6)*	
H72	−0.309 (3)	0.7796 (13)	0.1971 (19)	0.046 (6)*	
H73	−0.279 (3)	0.8637 (13)	0.2655 (19)	0.046 (6)*	
H81	0.309 (3)	0.7521 (12)	0.0095 (17)	0.036 (5)*	
H82	0.116 (2)	0.7740 (11)	−0.0672 (17)	0.029 (5)*	
H83	0.151 (2)	0.6936 (12)	0.0048 (15)	0.027 (5)*	
H111	0.153 (2)	1.0450 (11)	0.2285 (16)	0.029 (5)*	
H112	0.172 (2)	1.0293 (11)	0.1034 (17)	0.032 (5)*	

### Atomic displacement parameters (Å^2^)

**Table:**

	*U*^11^	*U*^22^	*U*^33^	*U*^12^	*U*^13^	*U*^23^
Cl1	0.0236 (2)	0.0411 (3)	0.0449 (3)	−0.01249 (17)	0.01322 (18)	−0.0198 (2)
O1	0.0232 (6)	0.0192 (5)	0.0174 (5)	0.0022 (4)	−0.0034 (4)	−0.0017 (4)
N1	0.0177 (6)	0.0145 (6)	0.0201 (6)	−0.0009 (4)	0.0047 (5)	−0.0012 (5)
N2	0.0240 (7)	0.0205 (6)	0.0159 (6)	−0.0001 (5)	0.0017 (5)	0.0010 (5)
C1	0.0180 (7)	0.0165 (7)	0.0196 (7)	0.0016 (5)	0.0059 (6)	−0.0023 (6)
C2	0.0162 (7)	0.0189 (7)	0.0177 (7)	0.0014 (5)	0.0051 (6)	0.0010 (5)
C3	0.0262 (8)	0.0226 (8)	0.0167 (7)	−0.0007 (6)	0.0074 (6)	0.0013 (6)
C4	0.0203 (7)	0.0194 (7)	0.0191 (7)	0.0041 (6)	0.0026 (6)	0.0026 (6)
C5	0.0162 (7)	0.0168 (7)	0.0184 (7)	−0.0009 (5)	0.0046 (6)	−0.0012 (5)
C6	0.0192 (7)	0.0157 (7)	0.0152 (7)	0.0023 (5)	0.0045 (6)	0.0001 (5)
C7	0.0201 (8)	0.0281 (9)	0.0335 (9)	−0.0020 (6)	0.0115 (7)	−0.0024 (7)
C8	0.0279 (9)	0.0247 (8)	0.0215 (8)	0.0045 (6)	0.0086 (7)	−0.0019 (6)
C9	0.0185 (7)	0.0146 (7)	0.0159 (7)	0.0008 (5)	0.0007 (5)	0.0003 (5)
C11	0.0230 (8)	0.0158 (7)	0.0267 (8)	−0.0008 (6)	0.0048 (6)	0.0015 (6)
C12	0.0239 (8)	0.0152 (7)	0.0253 (8)	−0.0020 (6)	0.0084 (6)	−0.0038 (6)
C13	0.0274 (9)	0.0496 (11)	0.0237 (9)	−0.0135 (8)	0.0093 (7)	−0.0002 (8)
C14	0.0277 (9)	0.0543 (12)	0.0217 (9)	−0.0116 (8)	0.0053 (7)	−0.0041 (8)
C15	0.0203 (7)	0.0236 (8)	0.0329 (9)	−0.0061 (6)	0.0108 (7)	−0.0114 (7)
C16	0.0287 (9)	0.0229 (8)	0.0379 (9)	−0.0015 (6)	0.0166 (8)	0.0046 (7)
C17	0.0271 (8)	0.0238 (8)	0.0294 (9)	0.0018 (6)	0.0087 (7)	0.0064 (7)

### Geometric parameters (Å, º)

**Table:**

Cl1—C15	1.7476 (16)	C6—C8	1.528 (2)
O1—C9	1.4175 (17)	C6—C9	1.538 (2)
O1—H1	0.87 (2)	C7—H71	1.00 (2)
N1—C11	1.4656 (19)	C7—H72	0.99 (2)
N1—C1	1.4698 (19)	C7—H73	0.98 (2)
N1—C2	1.4729 (19)	C8—H81	0.97 (2)
N2—C3	1.469 (2)	C8—H82	0.971 (19)
N2—C4	1.471 (2)	C8—H83	0.984 (19)
N2—H2	0.870 (19)	C9—H9	1.021 (18)
C1—C5	1.532 (2)	C11—C12	1.510 (2)
C1—H11	0.972 (16)	C11—H111	0.993 (19)
C1—H12	1.010 (18)	C11—H112	0.97 (2)
C2—C6	1.539 (2)	C12—C17	1.386 (2)
C2—H21	0.962 (17)	C12—C13	1.390 (2)
C2—H22	0.980 (17)	C13—C14	1.388 (2)
C3—C5	1.541 (2)	C13—H13	0.98 (2)
C3—H31	1.026 (19)	C14—C15	1.377 (2)
C3—H32	0.955 (18)	C14—H14	0.96 (2)
C4—C6	1.5381 (19)	C15—C16	1.376 (3)
C4—H41	1.014 (17)	C16—C17	1.397 (2)
C4—H42	1.005 (17)	C16—H16	0.95 (2)
C5—C7	1.530 (2)	C17—H17	0.97 (2)
C5—C9	1.530 (2)		
			
C9—O1—H1	106.2 (15)	C5—C7—H71	111.5 (12)
C11—N1—C1	110.47 (11)	C5—C7—H72	110.7 (12)
C11—N1—C2	110.08 (12)	H71—C7—H72	108.4 (17)
C1—N1—C2	111.27 (11)	C5—C7—H73	109.4 (12)
C3—N2—C4	111.30 (12)	H71—C7—H73	106.2 (17)
C3—N2—H2	109.3 (13)	H72—C7—H73	110.6 (18)
C4—N2—H2	104.8 (13)	C6—C8—H81	111.8 (12)
N1—C1—C5	112.58 (11)	C6—C8—H82	109.5 (11)
N1—C1—H11	106.9 (9)	H81—C8—H82	108.6 (16)
C5—C1—H11	109.4 (9)	C6—C8—H83	110.5 (11)
N1—C1—H12	111.0 (10)	H81—C8—H83	108.0 (16)
C5—C1—H12	109.4 (10)	H82—C8—H83	108.5 (15)
H11—C1—H12	107.3 (13)	O1—C9—C5	109.08 (11)
N1—C2—C6	113.11 (12)	O1—C9—C6	111.99 (12)
N1—C2—H21	107.7 (10)	C5—C9—C6	108.29 (11)
C6—C2—H21	110.0 (10)	O1—C9—H9	109.6 (10)
N1—C2—H22	109.7 (10)	C5—C9—H9	109.6 (10)
C6—C2—H22	109.8 (10)	C6—C9—H9	108.2 (10)
H21—C2—H22	106.2 (14)	N1—C11—C12	113.18 (12)
N2—C3—C5	115.77 (12)	N1—C11—H111	106.7 (11)
N2—C3—H31	107.9 (10)	C12—C11—H111	108.2 (11)
C5—C3—H31	107.9 (10)	N1—C11—H112	110.5 (11)
N2—C3—H32	106.1 (11)	C12—C11—H112	109.6 (11)
C5—C3—H32	110.9 (10)	H111—C11—H112	108.5 (15)
H31—C3—H32	108.0 (15)	C17—C12—C13	118.22 (15)
N2—C4—C6	114.89 (12)	C17—C12—C11	121.53 (15)
N2—C4—H41	108.1 (9)	C13—C12—C11	120.16 (14)
C6—C4—H41	109.2 (9)	C14—C13—C12	121.57 (16)
N2—C4—H42	108.7 (10)	C14—C13—H13	119.2 (12)
C6—C4—H42	109.4 (10)	C12—C13—H13	119.2 (12)
H41—C4—H42	106.2 (13)	C15—C14—C13	118.67 (17)
C7—C5—C9	111.12 (12)	C15—C14—H14	118.9 (13)
C7—C5—C1	108.97 (12)	C13—C14—H14	122.4 (13)
C9—C5—C1	108.28 (12)	C16—C15—C14	121.53 (16)
C7—C5—C3	108.52 (13)	C16—C15—Cl1	119.45 (13)
C9—C5—C3	108.21 (12)	C14—C15—Cl1	119.01 (14)
C1—C5—C3	111.76 (12)	C15—C16—C17	118.92 (16)
C8—C6—C4	108.87 (12)	C15—C16—H16	120.4 (15)
C8—C6—C9	111.18 (12)	C17—C16—H16	120.5 (14)
C4—C6—C9	107.90 (12)	C12—C17—C16	121.00 (16)
C8—C6—C2	108.67 (12)	C12—C17—H17	118.8 (13)
C4—C6—C2	111.71 (12)	C16—C17—H17	120.2 (13)
C9—C6—C2	108.54 (11)		
			
C11—N1—C1—C5	177.79 (12)	C3—C5—C9—C6	−60.28 (14)
C2—N1—C1—C5	55.20 (16)	C8—C6—C9—O1	−58.82 (15)
C11—N1—C2—C6	−176.75 (12)	C4—C6—C9—O1	−178.14 (11)
C1—N1—C2—C6	−53.93 (15)	C2—C6—C9—O1	60.65 (15)
C4—N2—C3—C5	−48.59 (17)	C8—C6—C9—C5	−179.14 (12)
C3—N2—C4—C6	49.63 (17)	C4—C6—C9—C5	61.55 (14)
N1—C1—C5—C7	179.66 (13)	C2—C6—C9—C5	−59.66 (14)
N1—C1—C5—C9	−59.36 (15)	C1—N1—C11—C12	157.40 (13)
N1—C1—C5—C3	59.73 (16)	C2—N1—C11—C12	−79.32 (16)
N2—C3—C5—C7	175.66 (13)	N1—C11—C12—C17	127.48 (16)
N2—C3—C5—C9	54.98 (16)	N1—C11—C12—C13	−56.2 (2)
N2—C3—C5—C1	−64.15 (16)	C17—C12—C13—C14	2.9 (3)
N2—C4—C6—C8	−177.88 (13)	C11—C12—C13—C14	−173.55 (17)
N2—C4—C6—C9	−57.11 (16)	C12—C13—C14—C15	−0.8 (3)
N2—C4—C6—C2	62.10 (17)	C13—C14—C15—C16	−2.1 (3)
N1—C2—C6—C8	177.65 (12)	C13—C14—C15—Cl1	177.03 (15)
N1—C2—C6—C4	−62.22 (16)	C14—C15—C16—C17	2.7 (3)
N1—C2—C6—C9	56.62 (15)	Cl1—C15—C16—C17	−176.40 (13)
C7—C5—C9—O1	58.55 (16)	C13—C12—C17—C16	−2.2 (2)
C1—C5—C9—O1	−61.09 (15)	C11—C12—C17—C16	174.16 (15)
C3—C5—C9—O1	177.60 (12)	C15—C16—C17—C12	−0.5 (3)
C7—C5—C9—C6	−179.33 (12)	C5—C9—O1—H1	−147.5 (15)
C1—C5—C9—C6	61.02 (14)	C6—C9—O1—H1	92.6 (15)

### Hydrogen-bond geometry (Å, º)

**Table:**

*D*—H···*A*	*D*—H	H···*A*	*D*···*A*	*D*—H···*A*
N2—H2···N1	0.870 (19)	2.287 (19)	2.8327 (18)	120.8 (16)
O1—H1···N2^i^	0.87 (2)	1.86 (2)	2.7242 (17)	172 (2)

Symmetry code: (i) *x*−1/2, −*y*+3/2, *z*−1/2.
